# Quantitative investigation of a novel small field of view hybrid gamma camera (HGC) capability for sentinel lymph node detection

**DOI:** 10.1259/bjr.20160435

**Published:** 2016-10

**Authors:** Mohammed S Alqahtani, John E Lees, Sarah L Bugby, Layal K Jambi, Alan C Perkins

**Affiliations:** ^1^ Space Research Centre, Department of Physics and Astronomy, University of Leicester, Leicester, UK; ^2^ Radiological Sciences Department, College of Applied Medical Sciences, King Khalid University, Abha, Saudi Arabia; ^3^ Radiological Sciences Department, College of Applied Medical Sciences, King Saud University, Riyadh, Saudi Arabia; ^4^ Radiological Sciences, School of Medicine, University of Nottingham, Nottingham, UK

## Abstract

**Objective:**

The hybrid gamma camera (HGC) has been developed to enhance the localization of radiopharmaceutical uptake in targeted tissues during surgical procedures such as sentinel lymph node (SLN) biopsy. To assess the capability of the HGC, a lymph node contrast (LNC) phantom was constructed to simulate medical scenarios of varying radioactivity concentrations and SLN size.

**Methods:**

The phantom was constructed using two clear acrylic glass plates. The SLNs were simulated by circular wells of diameters ranging from 10 to 2.5 mm (16 wells in total) in 1 plate. The second plate contains four larger rectangular wells to simulate tissue background activity surrounding the SLNs. The activity used to simulate each SLN ranged between 4 and 0.025 MBq. The activity concentration ratio between the background and the activity injected in the SLNs was 1 : 10. The LNC phantom was placed at different depths of scattering material ranging between 5 and 40 mm. The collimator-to-source distance was 120 mm. Image acquisition times ranged from 60 to 240 s.

**Results:**

Contrast-to-noise ratio analysis and full-width-at-half-maximum (FWHM) measurements of the simulated SLNs were carried out for the images obtained. Over the range of activities used, the HGC detected between 87.5 and 100% of the SLNs through 20 mm of scattering material and 75–93.75% of the SLNs through 40 mm of scattering material. The FWHM of the detected SLNs ranged between 11.93 and 14.70 mm.

**Conclusion:**

The HGC is capable of detecting low accumulation of activity in small SLNs, indicating its usefulness as an intraoperative imaging system during surgical SLN procedures.

**Advances in knowledge:**

This study investigates the capability of a novel small-field-of-view (SFOV) HGC to detect low activity uptake in small SLNs. The phantom and procedure described are inexpensive and could be easily replicated and applied to any SFOV camera, to provide a comparison between systems with clinically relevant results.

## INTRODUCTION

Radioguided surgery for sentinel lymph node biopsy (SLNB) is now a well-established technique in the staging of various cancers, with most surgeons using non-imaging gamma probes to locate the uptake of radioactivity in nodes.^1^ With existing detection approaches (gamma probes, pre-operative gamma camera imaging and the use of “blue dye”), a sensitivity rate of 95% and a false-negative rate of 5% have been reported for SLNB.^2,3^ Although this gives a relative high confidence for detection, enhancements in sensitivity and decreases in false-negative rates can be of benefit only to patients.

Intraoperative imaging has been suggested as a method to aid surgical localization of regions of radioactive uptake. The proposed demand for small-field-of-view (SFOV) gamma imaging systems, which can be brought into operating theatres, has led to the current development of a number of new intraoperative gamma cameras with widely varying designs and performance capabilities.^4–7^ A set of testing protocols has been suggested to provide a quantifiable comparison between these systems.^8^ However, the methods chosen for testing vary and do not always directly relate to the performance of a system in a true clinical situation. This article describes a new method for testing camera performance, providing information that is designed to be more intuitively understood by end-users.

**Figure 1.**
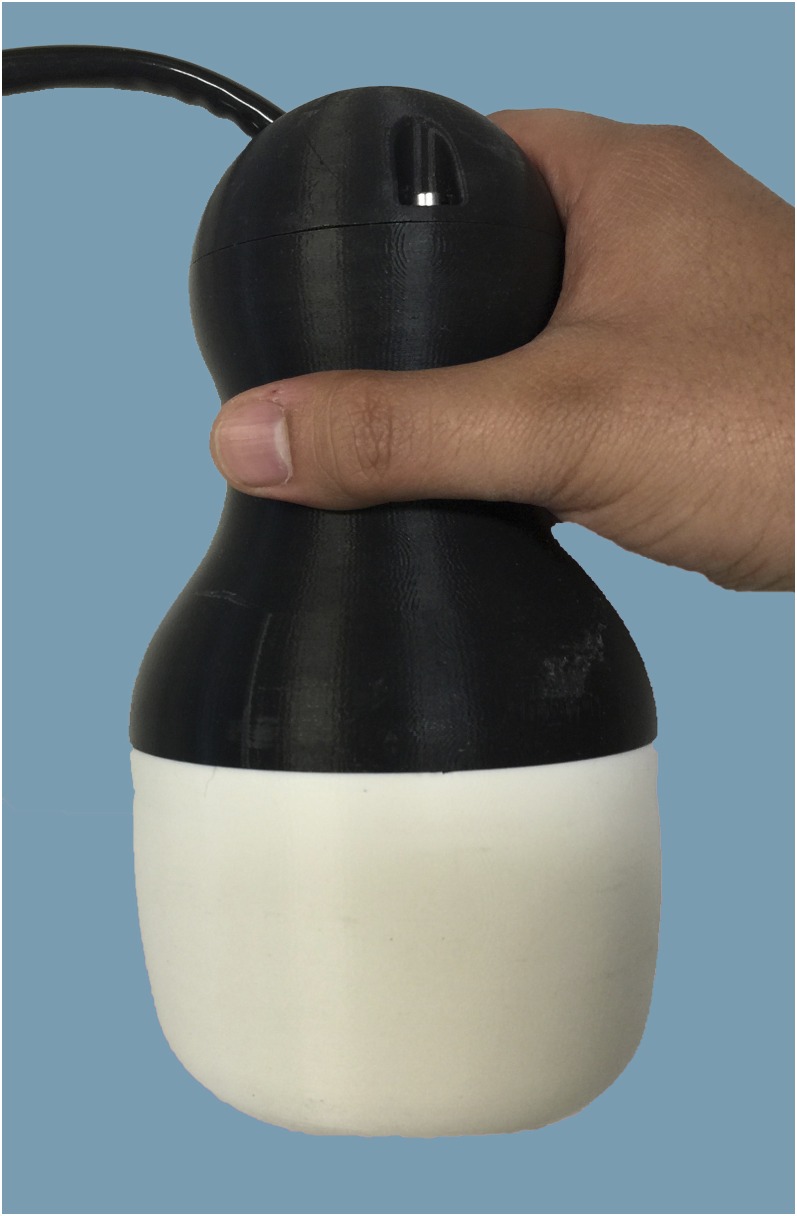
Photograph of the hybrid gamma camera.

To quantitatively evaluate the usefulness of the hybrid gamma camera (HGC) for scintigraphic imaging of patients during the SLNB procedure, a lymph node contrast (LNC) phantom has been designed and fabricated. The LNC phantom construction, acquisition procedure and activity simulation technique have been designed to simulate the clinical situation. The phantom has been used to assess the HGC produced by workers at the University of Leicester (Figure 1). The tests described could be applied to any intraoperative gamma camera or, with some adjustment, to non-imaging probes.

## METHODS AND MATERIALS

### Hybrid gamma camera

The hand-held SFOV HGC uses a scintillator-based detector. The detector consists of an e2v CCD97 back-illuminated electron-multiplying charge-coupled device coupled to a columnar CsI(Tl) scintillator Hamamatsu Photonics UK Limited, Welwyn Garden City, Hertfordshire, UK. The performance of the HGC was investigated with 600-μm-thick and 1500-μm-thick scintillators installed. The detector enclosure was shielded with tungsten (3-mm thick) on the sides to reduce the effect of scattered radiation. Imaging was carried out with a tungsten pinhole collimator of acceptance angle 60°, and the performance of the HGC was investigated with both a 0.5-mm-diameter and 1.0-mm-diameter pinhole collimators installed. The HGC also has the ability to provide fused optical gamma imaging (*i.e.* hybrid imaging) of the targeted features to improve localization. More details about the HGC design and structure have been provided elsewhere.^9–11^

### Lymph node contrast phantom construction

**Figure 2.**
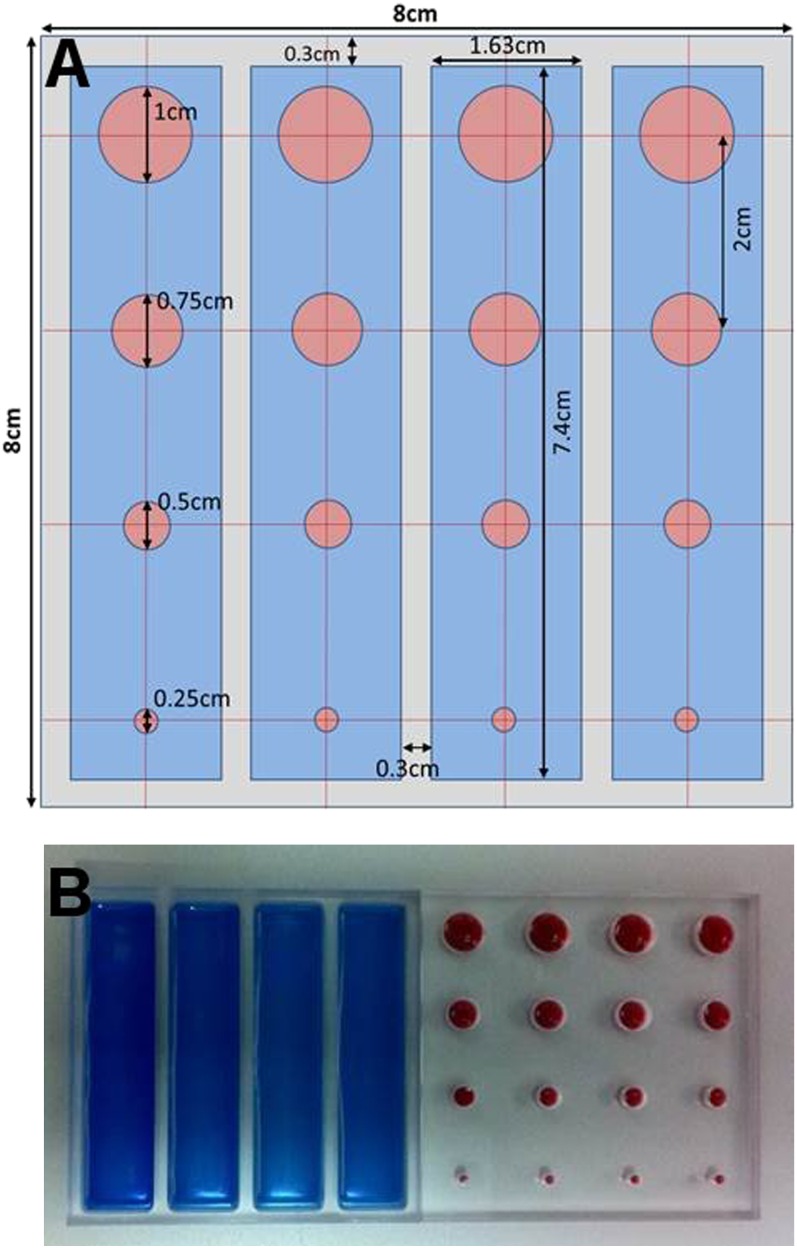
Schematic and photographs of the lymph node contrast (LNC) phantom: (a) a diagram of the LNC phantom dimensions and (b) a representation of the two Perspex plates used to construct the phantom.

The LNC phantom shown in Figure 2 consisted of two clear acrylic glass plates (80 × 80 mm); chemical formula: (C_5_O_2_H_8_)_*n*_ and density = 1.18 g cm^−3^. Acrylic glass is used for the construction of different types of medical phantoms owing to its suitable degree of similarity with body tissues.^12^ The first plate contained four rectangular wells of the same depth (6 mm). This plate (the background plate) was used to simulate the uptake of radioactivity in the tissues surrounding a feature of interest. The node plate had 4 groups of cylindrical wells of 2.5, 5, 7.5 and 10 mm diameter (6-mm depth); each group contains 4 wells (16 holes in total) in the arrangement shown in Figure 2a. The simulated sentinel lymph node (SLN) sizes were comparable with the majority of the SLNs inside the human body.^13^

The separate background and node plates allowed flexibility in phantom configuration. Nodes of different sizes could be compared against the same active background or nodes of the same size but differing activities could be compared (Figure 2b). Multiple data sets could be collected during a single acquisition period when the complete phantom was used. Scattering media could be added to the phantom to simulate deep-seated nodes. The phantom has been designed to be simple and inexpensive to construct.

### Radioactivity simulation

From a review of the literature, the largest reported ratio of SLN to background activity (NBR) when using ^99m^Tc was 1 : 10,^14^ and different activity concentrations were chosen to match this value. The radioactivity concentrations in the simulated SLNs were taken from the available medical data and are presented in Table 1. Further details about the activity simulation and data used for the scientific justification in these experiments have been extensively documented elsewhere.^15–18^

**Table 1.** Summary of the amount of ^99m^Tc activity used in the lymph node contrast phantom

Node diameter (mm)	Node volume (ml)	First row activity (MBq)		Second row activity (MBq)		Third row activity (MBq)		Fourth row activity (MBq)	
		Node	Background	Node	Background	Node	Background	Node	Background
10	0.4	4.0	3 in 3 ml	2.0	1.5 in 3 ml	1.0	0.75 in 3 ml	0.5	0.375 in 3 ml
7.5	0.2	2.0		1.0		0.5		0.25	
5	0.1	1.0		0.5		0.25		0.125	
2.5	0.02	0.2		0.1		0.05		0.025	

### Imaging procedure

The LNC phantom was placed beneath thicknesses of the scattering medium (*i.e.* clear acrylic glass plates), ranging between 5 and 40 mm. The distance between the HGC collimator and the simulated SLNs was 120 mm, chosen to provide a clinically useful field of view (FOV) of 90 ×90 mm. Acquisition time for each image was varied between 60 and 240 s, with 240 s taken as an upper limit on the length of time which would be appropriate to use intraoperatively. A Gaussian filter of width 2 pixels was applied to all images. Imaging sets were produced using 0.5-mm-diameter and 1.0-mm-diameter pinhole collimators and 600-μm-thick and 1500-μm-thick scintillators.

### Data analysis

**Figure 3.**
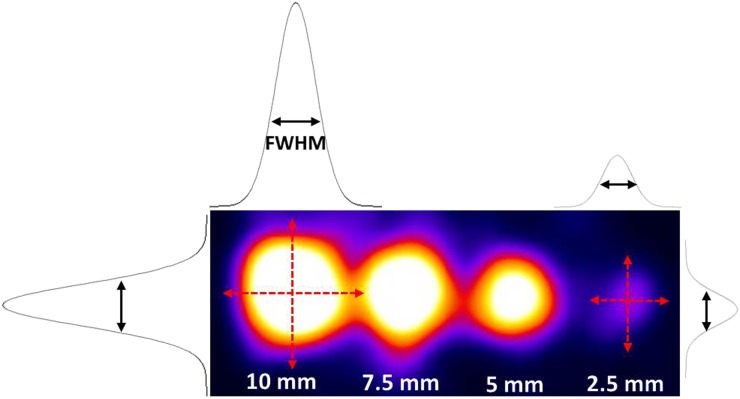
Illustration of node image size analysis. FWHM, full width at half maximum.

The imaging parameters measured were the size and the contrast-to-noise ratio (CNR) of each node. To calculate the imaged size of each node, 1-pixel-wide profiles were taken through the centre of the node in the vertical and horizontal directions (Figure 3). A Gaussian curve was fitted to each profile and the reported node sizes defined as the mean of the full-width-at-half-maximum (FWHM).

CNR is a parameter that was used to quantify the quality of images and so the ability of a specific medical imaging modality to distinguish between features and background.^19^ The detectability of a high activity feature, such as a node, in the presence of an active background depends on not only the contrast of the node but also its size and the level of background noise. CNR analysis allows all these factors to be considered in a single measurement.

**Figure 4.**
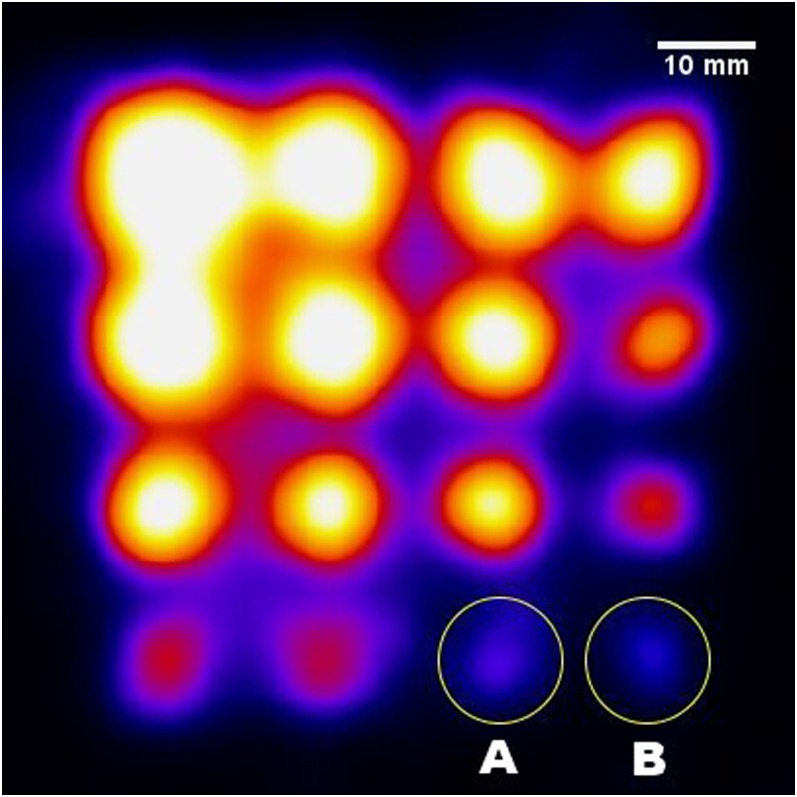
Gamma image for the lymph node contrast phantom while the simulated sentinel lymph nodes were located beneath 25 mm of scattering material; the hybrid gamma camera, while acquiring this image, was fitted with the 1500-μm-thick CsI(Tl) scintillator and the 1.0-mm pinhole collimator, and the acquisition time was 240 s. Circle (A): a node containing 0.05 MBq; circle (B): a node containing 0.025 MBq. The contrast-to-noise ratio values for node (A) and node (B) are 6.02 and 3.13, respectively.

Two regions of interest (ROIs) were required to calculate the CNR of a node. The node ROI was defined as a circular ROI centred on the node by eye, with the diameter set to the measured FWHM for that node for consistency.^20^ When nodes were not easily visible, sizes and positions were used from nodes of the same size with identical setups but higher node activities. The circular background ROI was positioned on a background region of the image (remote from all nodes), with a diameter double that of the node ROI. For larger nodes, this required an extra background well to be filled and imaged simultaneously to ensure that there was a large enough background region for measurement. The CNR was then calculated as

$$\text{CNR}\;=\;\left[\frac{\left(N_{\text{l}}-N_{\text{bg}}\right)}{\;\sigma_{\text{bg}}}\right]\text{,}$$

where *N*_l_ is the mean counts in the node ROI, *N*_bg_ is the mean counts in the background ROI and *σ*_bg_ is the standard deviation in background counts. To simplify and summarize the key results of the CNR analysis, two threshold CNR values have been set (3 and 5) based on Rose's^21^ approximation for detectability. Nodes with a CNR of 3 visually appeared barely detectable and required colour scaling to be clearly seen; nodes with a CNR of 5 could generally easily be seen in images without colour scaling, making these reasonable thresholds (Figure 4). In order to present the large amount of data collected, data sets were defined by the percentage of visible nodes (*i.e.* the percentage of nodes with CNR greater than the threshold).

## RESULTS

### Full-width-at-half-maximum measurements for simulated sentinel lymph nodes

The size of the imaged nodes was investigated with different thicknesses of scattering material, using different scintillator thicknesses and different pinhole sizes and with an acquisition time of 240 s. In all cases, a steady increase in the FWHM value was recorded when the scattering media thickness was increased. This reflects the expected degradation of spatial resolution due to scattered photons. In all cases, the detected size of the nodes was significantly smaller when imaged with the 0.5-mm pinhole rather than the 1.0-mm pinhole. This is an expected result due to the poorer spatial resolution of larger pinhole cameras.

**Figure 5.**
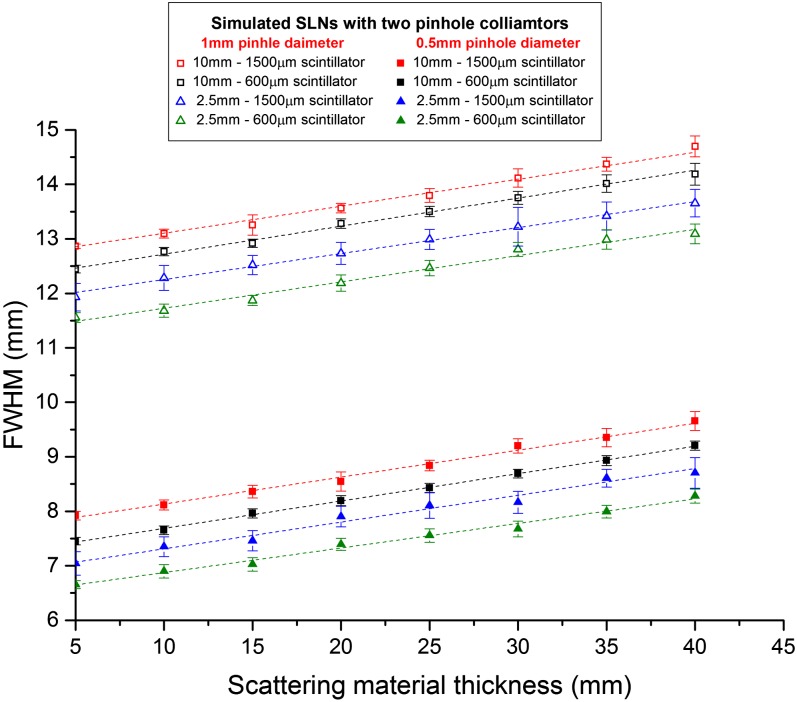
Full-width-at-half-maximum (FWHM) recorded values for the largest and smallest simulated sentinel lymph nodes (SLNs) (*i.e.* 2.5 and 10 mm in diameter); the simulated SLNs were imaged using the hybrid gamma camera fitted with 600-μm-thick and 1500-μm-thick CsI(Tl) scintillators and 0.5-mm-diameter and 1.0-mm-diameter pinhole collimators. The total imaging distance was 120 mm and the acquisition time was 240 s.

For the 600-µm-thick scintillator, the 2.5-mm node had a measured size of 6.65–8.27 mm (the calculated geometric resolution is 6.25 mm) for 5–40-mm node depths when imaged with the 0.5-mm-diameter pinhole; these increased to 11.56 and 13.10 mm when the 1.0-mm-diameter pinhole was fitted (Figure 5). The increase in measured node size, averaged over all four node sizes, from 5–40-mm depth was 23.88 and 17.23% using the 0.5-mm-diameter and 1.0-mm-diameter pinhole collimators, respectively. For the 1500-µm-thick scintillator, the measured sizes for the 2.5-mm node were 7.04–8.70 mm with the 0.5-mm-diameter pinhole and 11.93–13.66 mm with the 1.0-mm-diameter pinhole (Figure 5). The average variation in size measurements for the full range of depths was 21.96% for the 0.5-mm-diameter pinhole and 16.39% for the 1.0-mm pinhole.

Use of the thicker scintillator resulted in some resolution degradation; however, this was small (<6%) compared with the measured node sizes. This was a minimal change in resolution considering the increase in sensitivity expected when more than doubling the thickness of the scintillator. This preservation of spatial resolution was due to the light-guiding that is provided by the needle structure of the scintillation layer.^10^

### Contrast-to-noise ratio analysis for lymph node contrast phantom

To aid the analysis of the large number of individual values obtained, each data set in this section was defined as the percentage of imaged nodes which were detectable in the final image, based on a threshold CNR value defined by Rose's^21^ approximation. Figure 4 gives two examples of detected nodes at two different thresholds (*i.e.* 3 and 5). In this gamma image, 100% of the simulated SLNs are detectable at a threshold value of 3 and 93.75% of them are detectable at a threshold value of 5. For instance, nodes marked (A) and (B) are detectable with 6.02 and 3.13 CNR values, respectively (Figure 4). Both of these nodes could be visually recognized, suggesting that Rose's thresholds are a reasonable proxy for detectability in this case.

**Figure 6.**
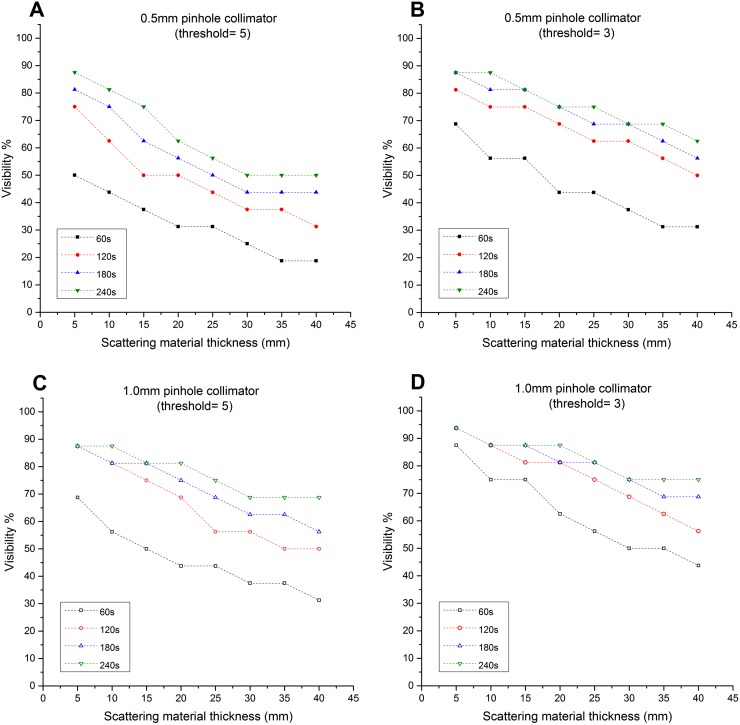
Graphs showing the relationship between the simulated sentinel lymph node (SLN) depths and the visibility (*i.e.* detection rate): these graphs represent the ability of the hybrid gamma camera fitted with the 600-μm-thick CsI(Tl) scintillator and two different pinhole collimators (*i.e.* 0.5-mm and 1.0-mm diameter) to detect the simulated SLNs at different threshold values (3 and 5).

**Figure 7.**
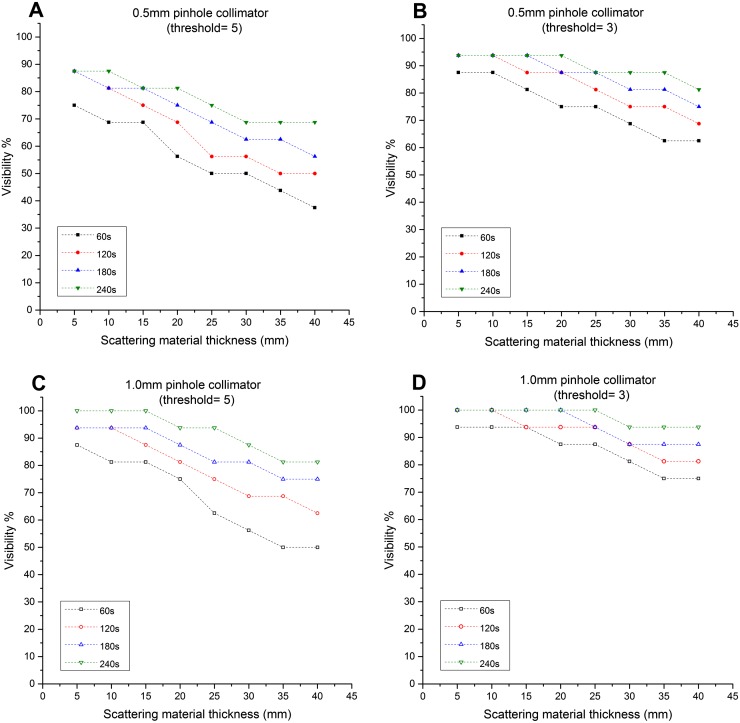
Graphs showing the relationship between the simulated sentinel lymph node (SLN) depths and the visibility (*i.e.* detection rate): these graphs represent the ability of the hybrid gamma camera fitted with the 1500-μm-thick CsI(Tl) scintillator and two different pinhole collimators (*i.e.* 0.5-mm and 1.0-mm diameter) to detect the simulated SLNs at different threshold values (3 and 5).

CNR results for the 600-µm-thick scintillator are shown in Figure 6 with those for the 1500-µm-thick scintillator shown in Figure 7. Detection levels were lowest for short acquisition times, for small and deep-seated nodes and when the smaller pinhole diameter was used, as would be expected. A large number of individual comparisons can be made and conclusions drawn from these data, and a selection of these is discussed in more detail below.

When the 600-µm-thick scintillator was installed, the HGC was not able to detect the smallest and weakest node tested (2.5-mm diameter, 25 kBq of ^99m^Tc) even under the most favourable conditions (1.0-mm pinhole, 5-mm depth, 240-s acquisition time). In a 240-s acquisition, with a 1.0-mm pinhole and the 1500-µm-thick scintillator installed, this node was detectable up to a depth of 25 mm for a threshold of 3 or 15 mm with a threshold of 5. Using the same experimental setup, with the HGC fitted with the 600-µm-thick scintillator, it was able to detect the 2.5-mm-diameter simulated SLN containing 100 kBq at both depths.

Results for the visibility of the simulated SLNs when they were located at depths between 10 and 40 mm and 60-s acquisition time with the CNR threshold set at 3 are presented in Table 2.

**Table 2.** Summary of the visibility for the simulated sentinel lymph nodes (SLNs) at 60-s acquisition time

SLN depth (mm)	Simulated SLN visibility (%)			
	600-μm-thick CsI(Tl) scintillator		1500-μm-thick CsI(Tl) scintillator	
	0.5-mm-diameter pinhole collimator	1.0-mm-diameter pinhole collimator	0.5-mm-diameter pinhole collimator	1.0-mm-diameter pinhole collimator
10	56.25	75	87.5	93.75
20	43.75	62.5	75	87.5
30	37.5	50	68.75	81.25
40	31.25	43.75	62.5	75

The detectability of the HGC is following the thickness of the scintillator used, and the diameter of the pinhole. While using the 600-µm-thick scintillator, and both pinhole collimators (*i.e.* 0.5-mm and 1.0-mm diameters), the recorded visibility was low compared with the 1500-µm-thick scintillator. For a 60-s acquisition time, the HGC had good detection rates for the simulated SLNs, varying between 93.75 and 75% with the gamma camera fitted with the 1.0-mm-diameter pinhole collimator and the 1500-µm-thick scintillator (Figure 7d). Using the same experimental setup and the 600-µm-thick scintillator, the detection rate degraded to 43.75% at 40-mm depth. However, using longer acquisition times improved the detection rate while using the 600-µm-thick scintillator. For instance, the detection rate at 40-mm depth is 75% at 240-s acquisition time (Figure 6d).

## DISCUSSION

Currently, the standard device that is used to detect radiopharmaceutical uptake in tissues intraoperatively is the non-imaging gamma probe. These non-imaging gamma probes are able to detect a very low accumulation of radioactivity (<10 kBq) in a short acquisition time (*i.e.* within seconds).^22,23^ However, gamma probes suffer from degradation of radial sensitivity when the targeted tissues are placed deeper than 20 mm, as the background signal may mask the targeted tissue signal.^22,23^ The HGC is able to distinguish various hot spots within the FOV with a superior spatial resolution, although currently it requires longer imaging times than non-imaging probes to detect smaller and weaker nodes. The HGC can also provide visual guidance with a relatively large FOV and is able to monitor and distinguish between different active anatomical structures such as SLNs within a limited area (*e.g.* Figure 4), which may override the benefit of a more sensitive gamma probe in some situations.^24^ Furthermore, hybrid images (*i.e.* fused optical gamma imaging) would enhance the accuracy of localization process during surgical procedures.^11,25^

This study shows the capability of the HGC to fulfil the majority of the requirements for a SFOV imaging device to be used for pre-operative, intraoperative and post-operative SLN investigations. The limits on HGC use will be due to sensitivity and the comparatively long acquisition times required, although for the activities investigated in this study, the majority of nodes were visible even with a 60-s acquisition time and a 40-mm node depth when the most ideal camera configuration was used.

This study indicates that of the two thicknesses used, the 1500-µm scintillator was the best choice for clinical use. With the thicker scintillator, the recorded CNR values were noticeably improved, and the HGC was able to detect the simulated SLNs with smaller diameters and lower activity accumulation. The 1500-μm-thick scintillator did have a poorer spatial resolution than the 600-μm-thick scintillator; however, the change in resolution (<6%) was relatively small compared with the change in detectability (>52% difference at 40-mm depth in 60-s acquisition time). The findings from this study suggest that an even thicker scintillator may be beneficial for SLN mapping and this needs to be investigated further.

For the majority of uses, particularly intraoperatively, the 1.0-mm-diameter pinhole is the appropriate choice, as its higher sensitivity greatly improves CNR at the cost of degraded spatial resolution. Unlike for scintillator thickness, however, this change is significant with resolution increasing by about 50% compared with the 0.5-mm pinhole. For this reason, for rapid sensitive imaging, as would be required intraoperatively, the 1.0-mm-diameter pinhole is preferred. Nevertheless, there are situations where there is no strict acquisition time limit or spatial resolution is of particularly importance, such as post-operative imaging, and in those cases, the operator may instead choose the 0.5-mm-diameter pinhole as more appropriate. The positioning of the camera and the location of the targeted features were also shown to affect detectability. Placing the head of the HGC as close as possible to the target area will improve both the spatial resolution and the sensitivity, with the sensitivity varying with the square of the distance from the radioactive source; however, the size of the FOV will be affected.

Some SFOV gamma camera systems have reported the ability to detect <7 kBq beneath up to 40 mm of scattering material in a short acquisition time (≤60 s).^26,27^ However, the low system spatial resolution provided by these SFOV gamma cameras will affect the progress of any intraoperative SLN detection procedure, *i.e.* even if these systems are able to detect the SLNs within a short acquisition time, they may require more time to take other views from various sides of the targeted area to distinguish the normal tissues from the abnormal tissues, particularly in situations where a SLN has been removed and the lymphatic vessels have been cut, with the possibility of more widespread distribution of the radioactivity within the surgical field.

## CONCLUSION

SLN detection procedures have grown together with an existing demand for developing medical procedures that enhance patient management and improve diagnosis processes. The HGC has been developed to provide additional localization information during procedures such as SLNBs.

In this study, an LNC phantom and an evaluation technique, which involved idealized physiological scenarios, were used to study the ability of the HGC to detect varying radioactivity concentrations and SLN sizes. Spatial resolution measurements and CNR analyses of the simulated SLNs were used as the main criteria to compare imaging sets produced by the HGC with acquisition times ranging between 60 and 240 s. The HGC could successfully detect 87.5–100% (acquisition times between 60 and 240 s) and 75–93.75% of the SLNs positioned beneath 20-mm and 40-mm thicknesses of scattering material, respectively. The results suggest that the most appropriate camera configuration for intraoperative SLN imaging was a 1500-µm-thick scintillator and a 1.0-mm-diameter pinhole.

The evaluation of the HGC in this study shows that it is well suited for SLN imaging. The capability of the HCG to detect low activity uptake in a small SLN indicates its usefulness as an intraoperative imaging system during critical surgical SLN procedures.
